# Supplementary material for: A Biochemical Platform to Define the Relative Specific Activity of IDUA Variants Identified by Newborn Screening
Source: Int J Neonatal Screen. 2020 Nov 12;6(4):88. doi: 10.3390/ijns6040088 (PMC7711455; doi:10.3390/ijns6040088)
Supplement: Supplementary file 1 [file IJNS-06-00088-s001.pdf]

Supplementary Figure 1

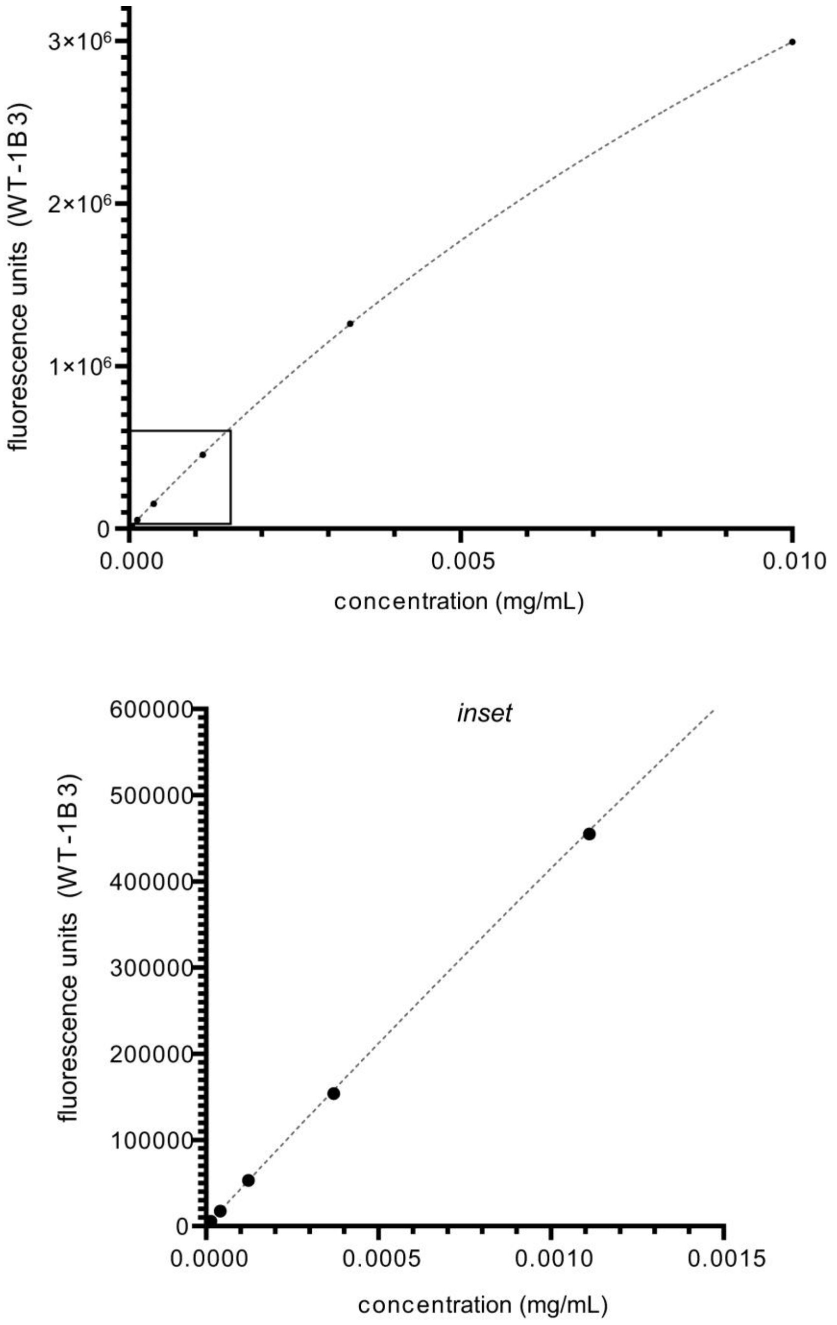

Standard curve for α-iduronidase activity in WT clone 1B3. Activity is shown as arbitrary fluorescence units and concentration of cell lysate as mg/mL. The box in the top panel is magnified and shown in the bottom panel.

Supplementary Figure 2

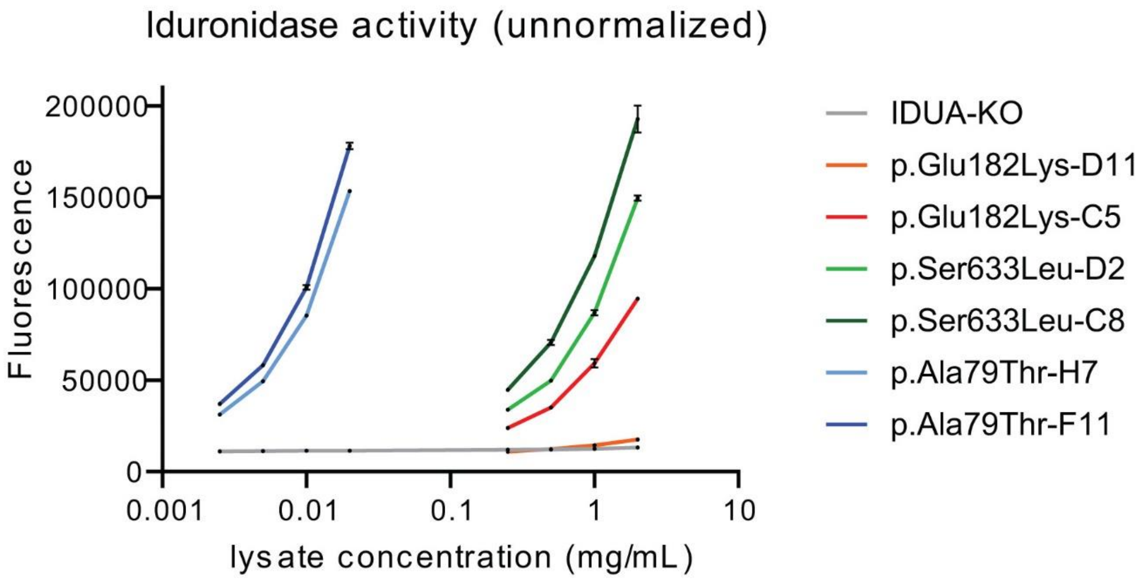

Activity of α-iduronidase was determined in several clones using a higher lysate concentration for more accurate stratification of residual activity.
